# Effects of Invasive Alien Plant Species on Native Plant Diversity and Crop Yield

**DOI:** 10.3390/plants13060888

**Published:** 2024-03-20

**Authors:** Kamil Najberek, Barbara Tokarska-Guzik, Damian Chmura, Wojciech Solarz

**Affiliations:** 1 Institute of Nature Conservation, Polish Academy of Sciences, Al. Adama Mickiewicza 33, 31-120 Kraków, Poland; solarz@iop.krakow.pl; 2 Faculty of Natural Sciences, Institute of Biology, Biotechnology and Environmental Protection, University of Silesia in Katowice, Jagiellońska 28, 40-032 Katowice, Poland; barbara.tokarska-guzik@us.edu.pl; 3 Institute of Environmental Protection and Engineering, University of Bielsko-Biala, Willowa Str 2, 43-309 Bielsko-Biala, Poland; dchmura@ubb.edu.pl

Invasive alien plant species may negatively affect the biological diversity of co-occurring native species, decrease the quality of invaded habitats, and even change the functioning of entire ecosystems [1,2]. These impacts can be induced or limited by various accompanying factors, such as climate change [3], and their scale may change over time and space, which contributes to difficulties in generalisations [4]. It is also known that biological invasions of alien plants have major impacts upon the human economy, including agricultural crops. Despite this, some crucial aspects of this phenomenon have rarely been investigated. For instance, it has been demonstrated that alien plants that are attractive to pollinators may significantly influence plant–pollinator interactions in invaded areas [5]. However, based on the studies available, it cannot be unequivocally concluded whether this is negative or positive (beneficial). For example, invasive alien *Impatiens* species may lure and co-opt pollinators of strawberries [6], whereas alien species occurring within fields of sunflowers may enhance the productivity of this crop [7]. The relevance and intensity of the influence of alien species may be species-specific or associated with a range of biotic and abiotic factors (pollinator communities in the study area, artificial or real field study conditions, etc.).

The seven original contributions to this issue of the *Plants* journal provide steps towards the improvement of knowledge of the causes and consequences of invasions by alien plant species for human use. The studied plants include some of the world’s most invasive alien species: Black Locust, *Robinia pseudoacacia*, Bohemian Knotweed, *Reynoutria ×bohemica*, Common Ragweed, *Ambrosia artemisiifolia*, Himalayan Balsam, *Impatiens glandulifera*, and Kudzu, *Pueraria montana*. Some of the aspects explored in the presented articles include below-ground phenomena, the role of mycorrhiza and allelopathic properties in the success of invasive alien plants, and the effects of these plants on soil properties.

Csiszár et al. explored the biodiversity-reducing effect of Black Locust, *Robinia pseudoacacia*, due to its nitrogen-fixing bacteria.

Levačić et al. confirmed that while allelopathy may play a significant role in the initial phases of invasion by Bohemian Knotweed, *Reynoutria ×bohemica*, it does not seem to contribute to its longer-term success in heavily invaded areas. This can be attributed to its efficient use of resources (light and nutrients) through which it outcompetes native plants.

Vagge and Chiaffarelli highlighted the role of high deterioration of the territorial landscape context in increasing the vulnerability of rice fields to invasions of alien plants, including the invasive *Murdannia keisak*.

Hall et al. demonstrated that extracts and residues of Common Ragweed, *Ambrosia artemisiifolia*, negatively affected the biomass and root production of soybean, wheat, and maize, as well as the performance of soil microorganisms.

Ab Razak et al. revealed that Himalayan Balsam, *Impatiens glandulifera*, increased its competitive abilities by limiting endophyte numbers and mycorrhizal colonisation in native plants, leading to an enhanced susceptibility to antagonists such as herbivorous insects.

Rusterholz et al. analysed the consequences of the escape and invasion of *Lamium galeobdolon argentatum* from gardens and public green space into forests, concluding that it reduced native plant species richness and altered soil properties.

Finally, Kato-Noguchi assessed the mechanisms and consequences of the multifaceted impacts exerted by one of the most globally invasive alien plants, *Pueraria montana* var. *lobata*. The results demonstrated that, due to its allelopathic properties, this species alters the composition of elements both in soil and aquatic environments. Moreover, the invasion of this plant results in an elevated emission of nitric oxide and isoprene into the local atmosphere and causes severe economic losses in agriculture and forestry.

Altogether, this collection of published articles reflects interest in one of the most significant problems affecting both nature conservation and food production: invasions by alien plant species. These contributions show that continued efforts are required in order to develop the scientific background and knowledge required to improve practical solutions for mitigating the negative consequences of alien plant introductions.

## Data Availability

Not applicable.
